# Association Between *ABCB1* Polymorphism and Stable Warfarin Dose Requirements in Brazilian Patients

**DOI:** 10.3389/fphar.2018.00542

**Published:** 2018-05-23

**Authors:** Letícia C. Tavares, Leiliane R. Marcatto, Renata A. G. Soares, Jose E. Krieger, Alexandre C. Pereira, Paulo C. J. L. Santos

**Affiliations:** ^1^Laboratory of Genetics and Molecular Cardiology, Heart Institute (InCor), Faculdade de Medicina FMUSP, Universidade de São Paulo, São Paulo, Brazil; ^2^Department of Pharmacology, Universidade Federal de São Paulo UNIFESP, São Paulo, Brazil

**Keywords:** warfarin pharmacogenetics, *ABCB1*, *MDR1*, *CYP4F2*, warfarin stable dose

## Abstract

The ideal dose of the oral anticoagulant warfarin varies widely among patients, mainly due to genetic factors. Genetic variations that impact warfarin pharmacokinetics and the vitamin K cycle are plausible candidates for being associated with warfarin dose requirements. Therefore, the aim of this study was to assess whether polymorphisms in the *ABCB1* and *CYP4F2* genes were associated with stable warfarin dose requirements in Brazilian patients. This retrospective study included samples from 309 individuals. Genotyping of *ABCB1* c.3435C>T and *CYP4F2* c.1297G>A were performed by polymerase chain reaction followed by melting curve analysis (HRM-PCR) and TaqMan® genotyping assay, respectively. Stable doses were adjusted in a linear multiple regression model for age, gender, body mass index, self-reported race, use of amiodarone, *CYP2C9* (^*^2 and ^*^3), *VKORC1* c.1639G>A, and *ABCB1* c.3435C>T or *CYP4F2* c.1297G>A. By performing a univariate analysis of variance, we found that the warfarin patients who carry *ABCB1* c.3435T variant alleles (CT and TT genotypes) need fewer warfarin stable doses in comparison with the individuals that are CC wild-type: 2.5 (*p* = 0.003) and 4.3 (*p* < 0.001) mg/week less, respectively, for the overall group of patients on stable anticoagulation therapeutics (*n* = 309); and 5.5 (*p* = 0.006) and 10.2 (*p* < 0.001) mg/week less, respectively, for the self-declared non-white stable subgroup (*n* = 76). No statistically significant differences in dose requirements were observed according to *CYP4F2* genotypes. In conclusion, our results suggest *ABCB1* c.3435C>T variant may influence warfarin dose requirements in Brazilian patients, when associated with other genotypic, demographic and clinical factors.

## Introduction

In the last 65 years, warfarin has been the most widely prescribed oral anticoagulant for treating and preventing thromboembolic events (Barnes et al., 2015). However, due to its narrow therapeutic index and high variance of pharmacokinetics and pharmacodynamics, warfarin interpatient responses are largely variable. Doses of warfarin administered out of the optimal therapeutic range commonly lead to serious adverse events, such as hemorrhagic and thromboembolic events in cases of over and under dosage, respectively. In addition to demographic and clinical factors, such as age, weight and use of concurrent medication, genetics is well established to play an important role in influencing warfarin maintenance dosage among patients (Klein et al., 2009; Johnson et al., 2011).

The key genetic variants that affect warfarin therapeutic response are presented in the *VKORC1* and *CYP2C9* genes, which encode the warfarin target enzyme and the primary warfarin metabolizer, respectively (Lee and Klein, 2013). However, as both these factors can explain 35–50% of the warfarin dose variability (Lee and Klein, 2013), there has been increasing interest in investigating additional clinically important genetic polymorphisms that may further explain interindividual warfarin dose variability among different populations.

Recently, studies have implicated the nonsynonymous variant presented in the *CYP4F2* gene (c.1297G>A, p.Val433Met, rs2108622) in warfarin sensitivity (Caldwell et al., 2008; Cooper et al., 2008; Borgiani et al., 2009; Takeuchi et al., 2009; Cen et al., 2010; Wei et al., 2012). The *CYP4F2* gene codes for the enzyme CYP4F2 (cytochrome P450 4F2), which is responsible for the primary metabolization of vitamin K in the liver. Warfarin mechanism of action consists of blocking the vitamin K redox cycle in order to diminish the concentration of the vitamin K reduced form, which is a coactivator of several coagulation factors (van Gorp and Schurgers, 2015). Thus, variations in the vitamin K bioavailability are speculated to interfere in the final response of treatment with warfarin.

Another genetic variant that has been investigated for influencing warfarin maintenance dosage is the silent polymorphism *ABCB1* c.3435C>T (rs1045642) (Wadelius et al., 2004; De Oliveira Almeida et al., 2011; Kim et al., 2013). The *ABCB1* gene (also called *MDR1*—*Multidrug Resistance 1 Gene*) codes for the well-known multidrug efflux pump P-glycoprotein (P-gp), responsible for restricting the absorption of many xenobiotics, as well as excreting them and their metabolites through the kidneys and liver. The referred *ABCB1* polymorphism has been extensively studied and associated with the variability dosage of many drugs, including warfarin, which is one of the many substrates of P-gp (Lepper et al., 2005; Salama et al., 2006).

In this context, the aim of this study was to assess the association of *ABCB1* and *CYP4F2* genotypes with warfarin dose requirements in Brazilian patients.

## Materials and methods

### Study population

This retrospective study included samples from 309 on stable warfarin treatment, derived from a cohort of 832 Brazilian patients enrolled between September/2011 and March/2012 at the Heart Institute (InCor), University of São Paulo Medical School (FMUSP), São Paulo, Brazil (Santos et al., 2015). The study protocol was approved by the Institutional Ethics Committee (Register Number 0804/10), and written informed consent was obtained from all participants prior to entering the study. Patients were categorized into self-declared “racial/color” subgroups, according to the Brazilian Census criteria, as White, Intermediate (meaning Brown, “Pardo” in Portuguese) or Black. The following exclusion criteria were considered: chronic liver failure, use of other anticoagulant drugs, receiving chemotherapy and referred alcoholism. Personal demographic and clinical data were obtained through a standardized interview with a pharmacist and checked out on electronic medical records (Santos et al., 2011a; Soares et al., 2012).

The length of time between INR tests was variable, depending on how long the patient was presenting an INR within the target range and the probability of events that might affect INR. In case of target range reached, the minimum considered period between one INR measure and another was 4 weeks. The maximum was 12 weeks, in case of the patient was taking VKA therapy with consistently stable INRs. These recommendations are in accordance with anticoagulant therapy management (Holbrook et al., 2012; Witt et al., 2016). Furthermore, over the period of stable INR, there were no changes of the recommended weekly dosage. Thus, we defined stable warfarin therapeutics as three consecutive INR measures within therapeutic range (1.8–3.2), with no need to adjust warfarin dosing.

### Genotyping

Genomic DNA was extracted from peripheral blood leukocytes by the standard salting-out method. Data of *VKORC1* c.-1639G>A (rs9923231), CYP2C9^*^2 (c.430C>T, rs1799853) and CYP2C9^*^3 (c.1075A>C, rs1057910) genotypes were obtained from the previous study of our group (Santos et al., 2015). Genotyping of *ABCB1* c.3435C>T (rs1045642) and *CYP4F2* p.Val433Met (rs2108622) were performed by polymerase chain reaction followed by melting curve analysis (HRM-PCR) and TaqMan® genotyping assay (Applied Biosystems, C__16179493_40), respectively, in a Rotor-Gene Q® (Qiagen) (Santos et al., 2011b). All included patients (*n* = 309) presented stable warfarin maintenance doses. Positive and negative controls were added in all experiments. Furthermore, as genotyping quality control, 6% of the samples were reanalyzed and gave 100% data consistency.

### Statistical analysis

For descriptive analysis purposes of the cohort characteristics, categorical variables are presented as percentages, and continuous variables are presented as mean ± standard deviation (SD) or standard error (SE) of the mean. Chi-square and Fisher's exact tests were performed for comparative analysis of categorical variables (such as Hardy–Weinberg equilibrium calculation) according to genotypes. One-way ANOVA (analysis of variance) test was used for analysis of warfarin stable maintenance dose according to *ABCB1* c.3435C>T (CC, CT or TT—categorized as 0, 1, and 2, respectively) and *CYP4F2* c.1297G>A (GG, GA or AA—categorized as 0, 1, and 2, respectively) genotypes.

Mean warfarin stable doses were adjusted through a linear multiple regression model for age (years), gender (1, if male, otherwise 0), Body Mass Index (BMI) (kg/m2), self-declared race (white, brown or black—categorized as 1, 2, and 3, respectively), amiodarone use (1, if the patient administers amiodarone, otherwise 0), predicted metabolic *CYP2C9* phenotypes (EM or IM + PM—categorized as 1 or 2, respectively), and *VKORC1* c.-1639G>A genotypes (GG, GA or AA—categorized as 0, 1, and 2, respectively). Tukey's HSD (honest significant difference) *post hoc* tests were performed to identify the different groups. The level of significance was set at *p* ≤ 0.05. All statistical analyses were carried out using SPSS statistical package (20.0 version) and R (3.4.4 version).

## Results

### General characteristics of the study cohort

Table 1 shows the demographic, clinical and genetic characteristics of the patients included in the study presenting stable warfarin treatment (*n* = 309). Of the 309 patients (mean age 64 ± 14), 154 (49.8%) were female, most of them (75.4%) declared their race/color as white, 4.6% were smokers and 10.7% used the warfarin concurrent medication amiodarone. The observed genotypic distributions for *VKORC1* and frequencies of *CYP2C9* predicted metabolic phenotypes are shown in Table 1. The observed required doses of warfarin ranged from 7.5 to 70 mg per week (median = 27.5, mean = 28.5 ± 11.0, in mg/week).

**Table 1:** General, clinical and genetic characteristics of the patients on stable warfarin therapeutics (*n* = 309).

**Variables**	**Value**
Gender, female (%)	49.8
Age (years)	64 ± 14
BMI (kg/m^2^)	27 ± 5
Smoking (%)	4.6
Amiodarone use (%)	10.7
Warfarin maintenance dose (mg/week)	28.5 ± 11.0
**SELF-DECLARED RACE/COLOR (%)**	
White	75.4
Brown	17.5
Black	7.1
***VKORC1*** **c.-1639G>A (%)**	
GG	49.5
GA	41.1
AA	9.4
***CYP2C9*** **PREDICTED METABOLIC PHENOTYPES (%)**	
EM	72.2
IM + PM	27.8

The presented genetic frequencies are regarding CYP2C9*2 (c.430C>T, rs1799853), CYP2C9*3 (c.1075A>C, rs1057910), and VKORC1 c.-1639G>A (rs9923231) polymorphisms. EM, Extensive metabolizer (*1/*1); IM, Intermediate metabolizer (*1/*2 or ^*^1/^*^3); PM, Poor metabolizer (*2/*2, *3/*3, or *2/*3). Continuous variables are expressed as mean ± standard deviation. Categorical variables are presented as frequencies, in percentage.

Supplementary Tables 1, 2 show the demographic, clinical and genetic characteristics of the stable patients according with *ABCB1* and *CYP4F2* genotypes, respectively (Supplementary Tables 1, 2).

### Frequencies of ABCB1 and CYP4F2 polymorphisms

Table 2 summarizes the frequencies of *ABCB1* and *CYP4F2* genotypes of the patients. For all included stable patients (*n* = 309), the frequencies of the T variant allele and of the homozygous genotype for the *ABCB1* c.3435C>T were 44 and 18.8%, respectively. The frequencies of the A variant allele and of the homozygous genotype for the *CYP4F2* c.1297G>A were 32 and 8.7%, respectively. *ABCB1* c.3435C>T and *CYP4F2* c.1297G>A genotypic distributions are in accordance with Hardy-Weinberg equilibrium (Table 2).

**Table 2:** Genotypic frequencies of *ABCB1* and *CYP4F2* polymorphisms of the patients with stable maintenance warfarin dose, according to self-declared racial subgroups (non-white or white).

**Polymorphism**	**Overall % (n) (*n* = 309)**	**Non-white % (n) (*n* = 76)**	**White % (n) (*n* = 233)**	*P*-value^*^
***ABCB1*** **c.3435C>T**				
CC	31.7 (98)	31.6 (24)	31.8 (74)	0.995
CT	49.5 (153)	50.0 (38)	49.4 (115)	
TT	18.8 (58)	18.4 (14)	18.8 (44)	
MAE (T allele)	44	43	44	
HWE chi-squared test (P)	0.899	0.8779	0.954	
***CYP4F2*** **c.1297G>A**				
GG	50.5 (156)	61.8 (47)	46.8 (109)	0.031
GA	40.8 (126)	35.5 (27)	42.5 (99)	
AA	8.7 (27)	2.7 (2)	10.7 (25)	
MAE (A allele)	32	20	32	
HWE chi-squared test (P)	0.828	0.412	0.722	

*HWE, Hardy-Weinberg equilibrium; MAE, minor allele frequency*.

*If HWE chi-squared test p < 0.05, there is no consistency with Hardy-Weinberg equilibrium*.

* *P-value of Chi-square and Fisher's Exact tests between genotypic frequencies of non-white vs. white subgroups for ABCB1 and CYP4F2 polymorphisms, respectively*.

### Mean warfarin dose according to *ABCB1* and *CYP4F2* genotypes

**Figure 1:**
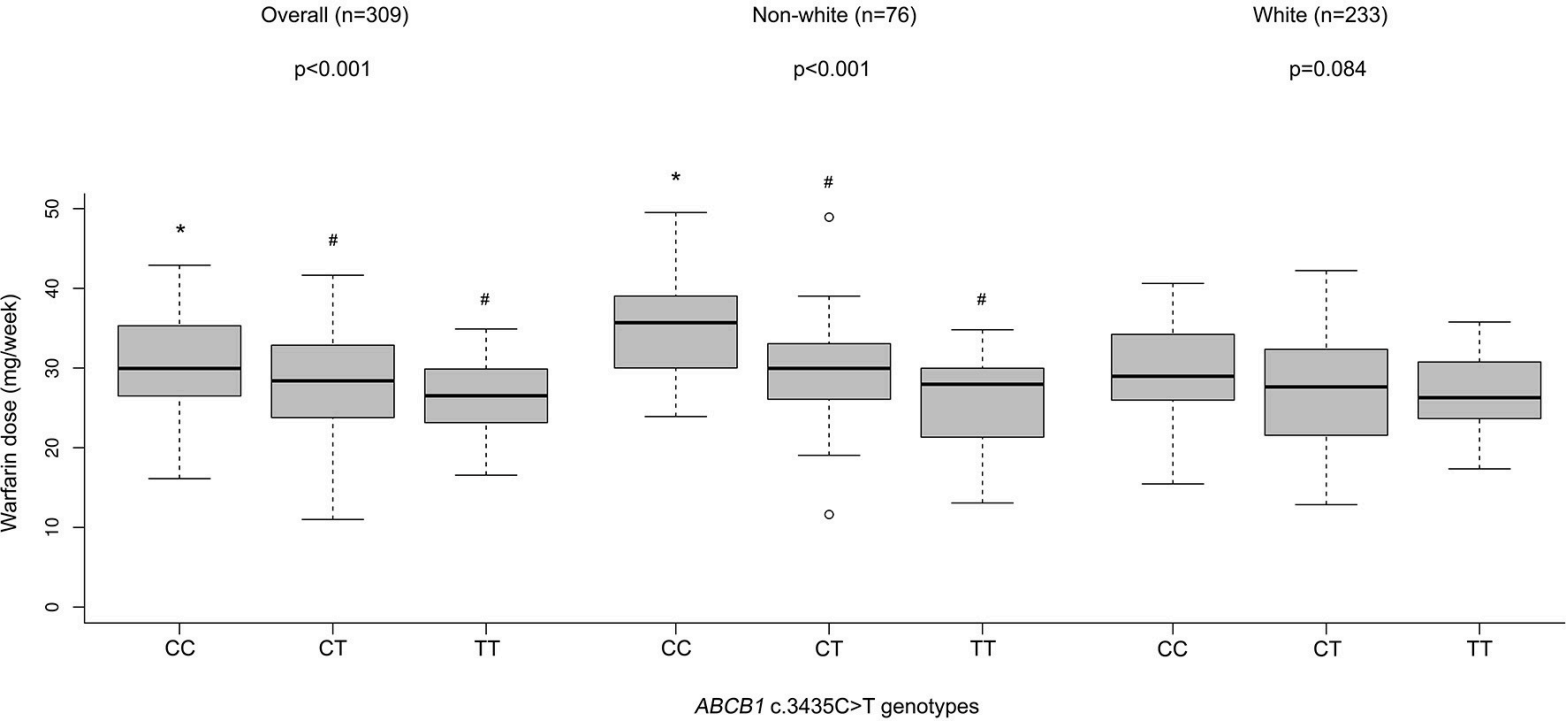
Warfarin dose (mg/week) according to *ABCB1* genotypes in patients with stable dose. Stable maintenance dose means three consecutive INR values within the target therapeutic range (1.8–3.2). Weekly warfarin stable doses were adjusted for age, gender, BMI, self-declared race (white, brown or black), amiodarone use, predicted metabolic *CYP2C9* phenotypes (EM or IM + PM), and *VKORC1* c.-1639G>A genotypes. For the white (*n* = 233) and non-white (*n* = 76) patient subgroups, self-declared race was not used as an independent variable. P values of ANOVA's test are presented. Values with different superscript symbols (*, #) are significantly different according to Tukey's HSD *post-hoc* test. Statistically significant differences were set when *p* < 0.05.

By performing a univariate analysis of variance (ANOVA) for the overall group of patients on stable warfarin treatment (*n* = 309), we found different mean warfarin doses according to *ABCB1* c.3435C>T genotypes *p*-value (ANOVA) < 0.001; CC: 30.5 ± 0.6 (*n* = 98), CT: 28.0 ± 0.5 (*n* = 153), TT: 26.2 ± 0.6 (*n* = 58) (mean stable dose ± SE in mg/week). A Tukey's HSD *post hoc* test revealed that patients with *ABCB1* 3435CC genotype presented higher doses than CT or TT carriers (CC vs. TT: *p* < 0.001; CC vs. CT: *p* = 0.003; CT vs. TT: *p* = 0.111). The data were adjusted for age, gender, BMI, self-reported race/color, use of amiodarone, *CYP2C9* (^*^2 and ^*^3), and *VKORC1* c.-1639G>A (Figure 1 and Table 3). The regression coefficients and respective p values are shown in Supplementary Tables 3, 4.

**Table 3:** Predicted warfarin doses by the multiple linear regression models adjusted with *ABCB1* genotypes for overall, white and non-white patients' subgroups.

**Subgroups**	***ABCB1* c.3435C>T genotypes**	**Mean predicted dose (mg/week)**	**SE**	**95% CI**		***P*-value**
Overall (*n* = 309)	CC	30.5	0.6	29.3	31.7	<0.001
	CT	28.0	0.5	27.1	29.0	
	TT	26.2	0.6	25.0	27.5	
Non-white (*n* = 76)	CC	35.3	1.3	32.6	38.0	<0.001
	CT	29.8	1.0	27.7	31.9	
	TT	25.1	2.0	20.8	29.3	
White (*n* = 233)	CC	30.0	0.7	27.5	30.4	0.084
	CT	27.3	0.6	26.1	28.5	
	TT	26.8	0.7	25.4	28.1	

Additionally, we separated patients with stable dose (*n* = 309) into two distinct subgroups according to self-declared race/color: white (*n* = 233) and non-white (*n* = 76). For this analysis, we adjusted the dose for all covariates cited above, except self-reported race. Table 2 summarizes the frequencies of *ABCB1* and *CYP4F2* genotypes of the patients with stable warfarin therapy according to self-declared racial subgroups (Table 2).

For the non-white subgroup, we still found differences in the mean warfarin doses according to *ABCB1* polymorphism *p*-value (ANOVA) < 0.001; CC = 35.3 ± 1.3 (*n* = 24), CT = 29.8 ± 1.0 (*n* = 38); TT = 25.1 ± 2.0 (*n* = 14) (mean stable dose ± SE in mg/week). Carriers of *ABCB1* 3435CC genotype presented higher doses than CT or TT, according to Tukey's HSD test (CC vs. TT: *p* < 0.001; CC vs. CT: *p* = 0.006; CT vs. TT: *p* = 0.063) (Figure 1 and Table 3).

Nevertheless, for the white subgroup, we have not found differences in the mean warfarin doses according to *ABCB1* polymorphism *p*-value (ANOVA) = 0.084; CC = 30.0 ± 0.7 (*n* = 74), CT = 27.3 ± 0.6 (*n* = 115); TT = 26.8 ± 0.7 (*n* = 44) (mean stable dose ± SE in mg/week) (Figure 1). P values according to Tukey's HSD test were: CC vs. TT: *p* = 0.126; CC vs. CT: *p* = 0.144; CT vs. TT: *p* = 0.867.

Regarding the *CYP4F2* p.Val433Met polymorphism, we found no significant statistical differences in the mean warfarin stable doses among the genotypic groups *p*-value (ANOVA) = 0.062; GG = 27.7 ± 0.4 (*n* = 156), GA = 29.3 ± 0.5 (*n* = 126), AA = 28.9 ± 0.9 (*n* = 27). *P*-values according to Tukey's HSD test were: GG vs. AA: *p* = 0.567; GG vs. GA: *p* = 0.053; GA vs. AA: *p* = 0.946. No significant differences were found when the white subgroup was assessed *p*-value (ANOVA) = 0.257; GG = 27.1 ± 0.6 (*n* = 109), GA = 28.3 ± 0.6 (*n* = 99), AA = 28.5 ± 1.1 (*n* = 25) (Supplementary Figure 1 and Supplementary Table 5).

For the non-white subgroup we found a slight difference in the mean warfarin doses according to *CYP4F2* polymorphism: *p*-value (ANOVA) = 0.048; GG = 29.2 ± 1.1 (*n* = 47), GA = 33.1 ± 1.0 (*n* = 27), AA = 33.0 ± 1.9 (*n* = 2). P values according to Tukey's HSD test were: GG vs. AA: *p* = 0.705; GG vs. GA: *p* = 0.042; GA vs. AA: *p* = 1.000. However, only two patients were *CYP4F2* 1297AA carriers and self-declared their race/color as non-white (Supplementary Figure 1 and Supplementary Table 5).

## Discussion

In the present study, we found the warfarin patients who carry *ABCB1* c.3435T variant alleles (CT and TT genotypes) need, on average, fewer warfarin stable maintenance doses in comparison to the individuals that are wild-type CC carriers. Similar data were obtained in the study of Wadelius et al. They found that an *ABCB1* haplotype containing only the 3435T allele as variant (called D haplotype) was over-represented [*p*(χ2) = 0.0242] among the low warfarin dose patients (<0.33 mg/kg, 60%) subgroup in comparison to the medium dose (0.33–0.46 mg/kg, 25%) and the high dose (>0.46 mg/kg, 15%) subgroups (*p* = 0.0094). On average, the patients that carried one 3435T variant allele for the D haplotype (*n* = 20) needed 24% lower warfarin dose and dose/body weight (BW) than the other patients (*n* = 181) who did not carry this haplotype (Wadelius et al., 2004).

In addition, Kim et al. found that patients with *ABCB1* c.3435TT genotypes needed, descriptively, the lowest dose of warfarin compared with those with the other 2 genotypes (warfarin dose (mg/week): CC: 59.5 (*n* = 70); CT: 56.9 (*n* = 90); TT: 55.6 (*n* = 36). Nevertheless, no statistical differences between the genotypic subgroups were found. However, according to race, it is possible to verify that the African, Hispanic, and Asian Americans (the non-white subgroup in Kim's study), when analyzed together presented a decreasing pattern of mean warfarin doses according to the addition of the 3435T allele (Kim et al., 2013). The authors considered that the study had little statistical power due to a small number of patients in some genotypic subgroups. Likewise, other studies have also failed to find statistically significant association between *ABCB1* c.3435C>T and warfarin dose requirements (Takeuchi et al., 2009; Issac et al., 2014).

Contrary to our result, a Brazilian study of De Oliveira Almeida et al. found that the *ABCB1* c.3435T variant allele contributes to higher warfarin doses in thrombophilic patients (De Oliveira Almeida et al., 2011; de Oliveira Almeida et al., 2014). This finding was exclusively seen for the subgroup of patients who needed up to 70 mg of weekly warfarin dose. It is valid to point out that in the study of De Oliveira Almeida et al. the frequency of the T allele was very high (66%) in comparison to the reported *ABCB1* c.3435C>T polymorphism frequency in other Brazilian studies, including the present one (around 40%) (Scheiner et al., 2010; Santos et al., 2011a). Furthermore, the patients were categorized into two subgroups: those receiving more and those receiving less than 70 mg warfarin weekly dose, which differs from the methodology of analysis of the present study.

In the literature, the studies that aimed to investigate the effect of the *ABCB1* c.3435C>T polymorphism on the codified P-glycoprotein (P-gp) are quite controversial (Fromm, 2002; Sakaeda et al., 2003; Lepper et al., 2005). Some studies have reported pronounced efflux of substrate and higher mRNA expression of P-gp in the gastrointestinal tract in 3435CC carriers in comparison to 3435CT or 345TT (Greiner et al., 1999; Hoffmeyer et al., 2000), while other studies have found that these parameters are significantly lower in the wild-type individuals (Moriya et al., 2002; Nakamura et al., 2002; Morita et al., 2003). In other words, some studies have observed that the *ABCB1* c.3435C>T polymorphism leads to augmented P-gp function or expression, although other studies have found that this variant causes decreased or loss of P-gp activity (Hoffmeyer et al., 2000; Hitzl et al., 2001; Kim et al., 2001; Sakaeda et al., 2003; Kimchi-Sarfaty et al., 2007). However, these studies were not performed with the same substrates and populations. So, it is likely that the effect of the *ABCB1* c.3435C>T polymorphism on the P-gp activity depends on the substrate-specificity and environmental factors (Perloff et al., 2001; Lepper et al., 2005).

Moreover, the biological assumptions about how the *ABCB1* c.3435C>T polymorphism affect P-gp are diverse. Despite the fact that this polymorphism is a silent mutation, as it does not lead to change of the codified isoleucine amino acid at position 1145, Kimchi-Sarfaty et al. have hypothesized that this variant may lead to a conformational P-gp change, which may affect the substrate specificity, explaining the observed affected efflux-pump function (Kimchi-Sarfaty et al., 2007). This conformational change could be due to distinct timing of cotranslational folding of the codon containing the variant. Furthermore, other so far unidentified variants that are in linkage disequilibrium with the c.3435C>T polymorphism, presented in control expression regions (e.g., promoter, enhancer) of the *ABCB1* gene, may influence P-gp expression (Hoffmeyer et al., 2000; Frittitta et al., 2001). Additionally, as the *ABCB1* c.3435C>T is in strong linkage disequilibrium with the non-synonymous c.2677T>G (p.Ser893Ala) and the synonymous c.1236T>C (p.Gly412=) variants, some authors hypothesize that the c.3435C>T itself does not directly influence the P-gp activity, but this common haplotype does (Salama et al., 2006; Saraeva et al., 2007).

We suggest that a possible biological explanation for our finding regarding the impact of *ABCB1* c.3435C>T polymorphism in the warfarin dose requirements is that the rs1045642 variant allele leads to an altered P-gp fold, affecting the warfarin site of recognition, and therefore leads to decreased warfarin efflux activity. It would cause augmented warfarin bioavailability and therefore lower warfarin doses would be required for those patients who are CT and TT carriers.

Our study presents the following limitation: we did not assess the genetic ancestry of the patients. Instead, we used the self-declared race/color parameter, categorized according to the Brazilian Census IBGE (Brazilian Institute of Geography and Statistics) criteria. Self-declared race/color does not correlate completely with genetic ancestry in Brazilians (Pena et al., 2011), as the self-perception of color/race in Brazil is primarily accounted for by phenotypic characteristics, as well as socio, cultural and geographic factors into which the subject is inserted (Santos et al., 2009). However, this parameter is useful in the real-life setting.

The association between *ABCB1* c.3435C>T and warfarin dose requirements, the main finding of this study, was observed for the overall stable group and non-white self-declared subgroup of patients, but not for those who self-declared their race/color as white. So, the above cited limitation might be, at least partially, contributing to this lack of association. The c.3435T variant allele in the *ABCB1* gene is frequently rarer in brown and black than in white people (Santos et al., 2011a; Soares et al., 2012). However, in the study of Kim et al. the P-gp function was found to be similar, on average, in European Americans and African Americans, for example (Kim et al., 2001). Thus, further studies are needed to elucidate the specific impact of the *ABCB1* c.3435C>T polymorphism in non-whites.

Regarding the lack of association of the *CYP4F2* c.1297G>A polymorphism with warfarin dose requirements in the present study, when this variant was included as a covariate in a multiple-regression analysis along with other relevant clinical (age, gender, BMI, self-declared race, amiodarone use) and genetic factors (*VKORC1* and *CYP2C9* genotypes), it did not contribute significantly to the model, even when self-declared race/color subgroups were assessed. Nevertheless, we verified a descriptive bias of augmented dose in individuals that carry the polymorphic allele A, but with no statistical significance (corresponding to GA and AA genotypes): GG = 27.7 ± 0.4 (*n* = 156), GA = 2 9.3 ± 0.5 (*n* = 126), AA = 28.9 ± 0.9 (*n* = 27).

Through a genome wide association study (GWAS), Caldwell et al. implicated the *CYP4F2* c.1297G>A polymorphism as a minor significant influencer of warfarin dose variability. The authors found that in three independent cohorts of white patients who had a stable warfarin dose (*n* = 1,009), the *CYP4F2* c.1297G>A variant contributed to approximately 1 mg/day more in homozygous AA individuals, when compared to wild-type (Caldwell et al., 2008). Other studies reported similar results (Zhang et al., 2009; Perini et al., 2010; Botton et al., 2011).

In conclusion, our results suggest *ABCB1* c.3435C>T polymorphism may marginally influence warfarin dose requirements in Brazilian patients, when associated with other genotypic, demographic and clinical factors.

## Ethics statement

This study was approved by the ethics committee and all included subjects provided written informed consent (SDC: 4519/17/019, CAPPesq: 0804/10).

## Author contributions

All authors have contributed substantially to the conception and design of this paper. LT performed the experiments, collected and analyzed the data, and wrote the paper. LM recruited the patients, collected data and critically revised the manuscript. RS recruited the patients. JK and AP provided the facilities. PS analyzed the data and critically revised the manuscript.

### Conflict of interest statement

The authors declare that the research was conducted in the absence of any commercial or financial relationships that could be construed as a potential conflict of interest.
