# Supplementary material for: Association Between ABCB1 Polymorphism and Stable Warfarin Dose Requirements in Brazilian Patients
Source: Front Pharmacol. 2018 May 23;9:542. doi: 10.3389/fphar.2018.00542 (PMC5975540; doi:10.3389/fphar.2018.00542)
Supplement: Supplementary file 1 [file Data_Sheet_1.PDF]

Supplementary Material

Association of *ABCB1* polymorphism with stable warfarin dose requirements in Brazilian patients

Letícia C Tavares, Leiliane R Marcatto, Renata A G Soares, Jose E Krieger, Alexandre C Pereira, Paulo C J L Santos*

*** Correspondence:**
Dr. Paulo Caleb Junior Lima Santos
paulo.caleb@unifesp.br

SUPPLEMENTARY FIGURES AND TABLES

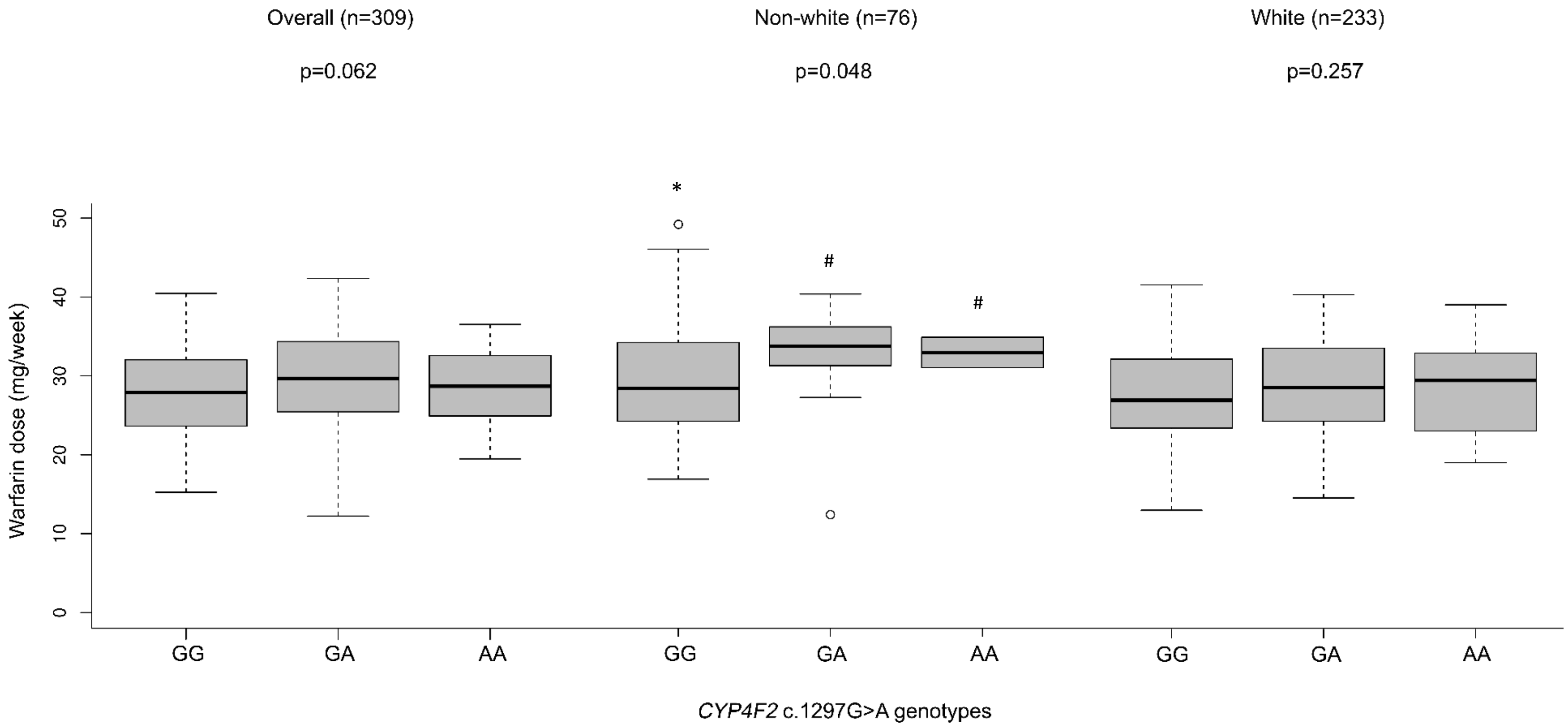

Supplementary Figure 1. Warfarin dose (mg/week) according to *CYP4F2* genotypes in patients with stable dose. Stable maintenance dose means three consecutive INR values within the target therapeutic range (1.8 to 3.2). Weekly warfarin stable doses were adjusted for age, gender, BMI, self-declared race (white, brown or black), amiodarone use, predicted metabolic *CYP2C9* phenotypes (EM or IM + PM), and *VKORC1* c.-1639G>A genotypes. For the white (n=233) and non-white (n=76) patient subgroups, self-declared race was not used as an independent variable. P values of ANOVA's test are presented. Values with different superscript symbols (*, #) are significantly different according to Tukey's HSD *post hoc* test. Statistically significant differences were set when $p<0.05$.

Supplementary Table 1. General, clinical and genetic characteristics of the patients according to *ABCB1* c.3435C>T genotypes (n=309).

Variables	*ABCB1* c.3435C>T genotypes			P value
	CC (n=98)	CT (n=153)	TT (n=58)	
**Gender, female (%)**	44.9 (44)	52.3 (80)	51.7 (30)	0.495
**Age (years)**	63 ± 15	64 ± 11	64 ± 14	0.816
**BMI (kg/m²)**	26 ± 5	27 ± 5	27 ± 4	0.134
**Self-declared race/color (%)**				
**White**	75.5 (74)	75.2 (115)	75.9 (44)	1.000
**Brown**	17.4 (17)	17.6 (27)	17.2 (10)	
**Black**	7.1 (7)	7.2 (11)	6.9 (4)	
**Smoking (%)**	7.1 (7)	3.9 (6)	1.7 (1)	0.329
**Amiodarone use (%)**	14.3 (14)	9.2 (14)	8.6 (5)	0.406
***VKORC1* c.-1639G>A (%)**				
**GG**	53.1 (52)	46.4 (71)	51.7 (30)	0.645
**GA**	39.8 (39)	41.8 (64)	41.4 (24)	
**AA**	7.1 (7)	11.8 (18)	6.9 (4)	
***CYP2C9* predicted metabolic phenotypes (%)**				
**EM**	70.4 (69)	72.5 (111)	74.1 (43)	0.872
**IM + PM**	29.6 (29)	27.5 (42)	25.9 (15)	

The presented genetic frequencies are regarding *CYP2C9*2* (c.430C>T, rs1799853), *CYP2C9*3* (c.1075A>C, rs1057910) and *VKORC1* (c.-1639G>A, rs9923231) polymorphisms. EM: Extensive metabolizer (*1/*1); IM: Intermediate metabolizer (*1/*2 or *1/*3); PM: Poor metabolizer (*2/*2, *3/*3, or *2/*3). Continuous variables are expressed as mean ± standard deviation. Categorical variables are presented as frequencies, in percentage. P value of Chi-square, Fisher's Exact or ANOVA tests for the *ABCB1* genotypic subgroups, depending on the analyzed variable.

Supplementary Table 2. General, clinical, and genetic characteristics of the patients according to *CYP4F2* c.1297C>T genotypes (n=309).

Variables	*CYP4F2* c.1297G>A genotypes			P value
	GG (n=156)	GA (n=126)	AA (n=27)	
**Gender, female (%)**	47.4 (74)	53.2 (67)	48.1 (13)	0.653
**Age (years)**	64 ± 13	63 ± 15	64 ± 14	0.902
**BMI (kg/m²)**	27 ± 5	27 ± 4	27 ± 4	0.997
**Self-declared race/color (%)**				
**White**	69.9 (109)	78.6 (99)	92.6 (25)	0.040
**Brown**	19.2 (30)	17.5 (22)	7.4 (2)	
**Black**	10.9 (17)	4.0 (5)	0.0 (0)	
**Smoking (%)**	5.3 (8)	4.8 (6)	3.7 (1)	0.769
**Amiodarone use (%)**	12.2 (19)	10.3 (13)	3.7 (1)	0.469
***VKORC1* c.-1639G>A (%)**				
**GG**	46.2 (72)	55.6 (70)	40.7 (11)	0.235
**GA**	44.9 (70)	36.5 (46)	40.7 (11)	
**AA**	9.0 (14)	7.9 (10)	18.5 (5)	
***CYP2C9* predicted metabolic phenotypes (%)**				
**EM**	73.1 (114)	70.6 (89)	74.1 (20)	0.878
**IM + PM**	26.9 (42)	29.4 (37)	25.9 (7)	

The presented genetic frequencies are regarding *CYP2C9*2* (c.430C>T, rs1799853), *CYP2C9*3* (c.1075A>C, rs1057910) and *VKORC1* (c.-1639G>A, rs9923231) polymorphisms. EM: Extensive metabolizer (*1/*1); IM: Intermediate metabolizer (*1/*2 or *1/*3); PM: Poor metabolizer (*2/*2, *3/*3, or *2/*3). Continuous variables are expressed as mean ± standard deviation. Categorical variables are presented as frequencies, in percentage. P value of Chi-square, Fisher's Exact or ANOVA tests for the *ABCB1* genotypic subgroups, depending on the analyzed variable.

Supplementary Table 3. Multiple linear regression models adjusted with *ABCB1* genotypes, for overall, white and non-white subgroups of patients on warfarin stable therapeutics.

Covariates	Overall (n=309)		Non-white (n=76)		White (n=233)	
	**β**	**P value**	**β**	**P value**	**β**	**P value**
**Intercept**	40.547	<0.001	46.859	<0.001	34.833	<0.001
***ABCB1* c.3435C>T**	-2.285	0.003	-4.762	0.006	-1.606	0.058
***CYP2C9* (*2 and *3)**	-2.596	0.032	2.849	0.289	-4.344	0.001
***VKORC1* c.-1639G>A**	-6.394	<0.001	-3.353	0.063	-7.371	<0.001
**Age**	-0.189	<0.001	-0.312	0.001	-0.121	0.006
**Gender**	1.028	0.344	3.209	0.176	0.455	0.699
**BMI**	0.318	0.007	0.156	0.507	0.473	<0.001
**Amiodarone use**	-7.823	<0.001	-11.424	0.002	-6.277	0.001
**Self-declared race/color**	0.649	0.482	-	-	-	-

Weekly stable dose was used as dependent variable. β is the regression coefficient. Covariates were categorized as follow: age (year), gender (1, if male, otherwise 0), body mass index (BMI) (kg/m^2), self-declared race (white, brown or black – categorized as 1, 2 and 3, respectively), amiodarone use (1, if the patient administers amiodarone, otherwise 0), predicted metabolic *CYP2C9* phenotypes (EM or IM + PM – categorized as 1 or 2, respectively), *VKORC1* c.-1639G>A genotypes (GG, GA or AA – categorized as 0, 1 and 2, respectively) and *ABCB1* c.3435C>T genotypes (CC, CT or TT – categorized as 0, 1 and 2, respectively).

Supplementary Table 4. Multiple linear regression models adjusted with *CYP4F2* genotypes, for overall, white and non-white subgroups of patients on warfarin stable therapeutics.

Covariates	Overall (n=309)		Non-white (n=76)		White (n=233)	
	**β**	**P value**	**β**	**P value**	**β**	**P value**
**Intercept**	38.113	<0.001	44.703	<0.001	32.668	<0.001
***CYP4F2* c.3435C>T**	0.897	0.298	1.185	0.612	1.099	0.215
***CYP2C9* (*2 and *3)**	-2.534	0.039	1.771	0.528	-4.159	0.002
***VKORC1* c.-1639G>A**	-6.403	<0.001	-3.062	0.116	-7.448	<0.001
**Age**	-0.190	<0.001	-0.325	0.001	-0.119	0.007
**Gender**	1.227	0.265	4.091	0.101	0.575	0.628
**BMI**	0.302	0.012	0.120	0.629	0.460	0.001
**Amiodarone use**	-7.374	<0.001	-10.926	0.007	-5.877	0.003
**Self-declared race/color**	0.801	0.400	-	-	-	-

Weekly stable dose was used as dependent variable. β is the regression coefficient. Covariates were categorized as follow: age (year). gender (1. if male. otherwise 0). body mass index (BMI) (kg/m²). self-declared race (white. brown or black – categorized as 1. 2 and 3. respectively). amiodarone use (1. if the patient administers amiodarone. otherwise 0). predicted metabolic *CYP2C9* phenotypes (EM or IM + PM – categorized as 1 or 2. respectively). *VKORC1* c.-1639G>A genotypes (GG. GA or AA – categorized as 0. 1 and 2. respectively) and *CYP4F2* c.1297G>A genotypes (GG. GA or AA – categorized as 0. 1 and 2. respectively).

Supplementary Table 5. Predicted warfarin doses by the multiple linear regression models adjusted with *CYP4F2* genotypes, for overall, white and non-white subgroups.

<table>
<tr><th>Subgroups</th><th>CYP4F2 c.1297G>A genotypes</th><th>Mean predicted dose (mg/week)</th><th>SE</th><th colspan="2">95% CI</th><th>P value</th></tr>
<tr><td colspan="7"></td></tr>
<tr><td rowspan="3">Overall (n=309)</td><td>GG</td><td>27.7</td><td>0.4</td><td>26.8</td><td>28.6</td><td rowspan="3">0.062</td></tr>
<tr><td>GA</td><td>29.3</td><td>0.5</td><td>28.2</td><td>30.3</td></tr>
<tr><td>AA</td><td>28.9</td><td>0.9</td><td>27.0</td><td>30.8</td></tr>
<tr><td colspan="7"></td></tr>
<tr><td rowspan="3">Non-white (n=76)</td><td>GG</td><td>29.2</td><td>1.1</td><td>27.1</td><td>31.3</td><td rowspan="3">0.048</td></tr>
<tr><td>GA</td><td>33.1</td><td>1.0</td><td>31.0</td><td>35.2</td></tr>
<tr><td>AA</td><td>33.0</td><td>1.9</td><td>8.4</td><td>57.5</td></tr>
<tr><td colspan="7"></td></tr>
<tr><td rowspan="3">White (n=233)</td><td>GG</td><td>27.1</td><td>0.6</td><td>25.9</td><td>28.2</td><td rowspan="3">0.257</td></tr>
<tr><td>GA</td><td>28.3</td><td>0.6</td><td>27.1</td><td>29.5</td></tr>
<tr><td>AA</td><td>28.5</td><td>1.1</td><td>26.2</td><td>30.9</td></tr>
</table>
